# Enhancing Aboveground Biomass Prediction through Integration of the SCDR Paradigm into the U-Like Hierarchical Residual Fusion Model

**DOI:** 10.3390/s24082464

**Published:** 2024-04-11

**Authors:** Ruofan Zhang, Jialiang Peng, Hailin Chen, Hao Peng, Yi Wang, Ping Jiang

**Affiliations:** 1 College of Information and Intelligence, Hunan Agricultural University, Changsha 410128, China; 2 College of Mechanical and Electrical Engineering, Hunan Agricultural University, Changsha 410128, China

**Keywords:** deep learning, biomass prediction, deep regression

## Abstract

Deep learning methodologies employed for biomass prediction often neglect the intricate relationships between labels and samples, resulting in suboptimal predictive performance. This paper introduces an advanced supervised contrastive learning technique, termed Improved Supervised Contrastive Deep Regression (SCDR), which is adept at effectively capturing the nuanced relationships between samples and labels in the feature space, thereby mitigating this limitation. Simultaneously, we propose the U-like Hierarchical Residual Fusion Network (BioUMixer), a bespoke biomass prediction network tailored for image data. BioUMixer enhances feature extraction from biomass image data, facilitating information exchange and fusion while considering both global and local features within the images. The efficacy of the proposed method is validated on the Pepper_Biomass dataset, which encompasses over 600 original images paired with corresponding biomass labels. The results demonstrate a noteworthy enhancement in deep regression tasks, as evidenced by performance metrics on the Pepper_Biomass dataset, including *RMSE* = 252.18, *MAE* = 201.98, and *MAPE* = 0.107. Additionally, assessment on the publicly accessible GrassClover dataset yields metrics of *RMSE* = 47.92, *MAE* = 31.74, and *MAPE* = 0.192. This study not only introduces a novel approach but also provides compelling empirical evidence supporting the digitization and precision improvement of agricultural technology. The research outcomes align closely with the identified problem and research statement, underscoring the significance of the proposed methodologies in advancing the field of biomass prediction through state-of-the-art deep learning techniques.

## 1. Introduction

In the face of global challenges such as population growth, climate change, and threats to food security, the adoption of digitalization and precision agriculture has become essential for the current and future development of agriculture. A research report indicates that relying solely on existing agricultural technologies may be inadequate to meet the increasing food demands projected for 2050 [1]. Aboveground biomass (AGB), a critical physiological parameter influencing crop growth and development, serves as a key indicator for assessing crop health and productivity. It plays a crucial role in various field management practices, including fertilizer application, weed and pest control, and exhibits a close correlation with crop yield [2,3,4]. Biomass, encompassing the total organic matter within a defined area over a specific timeframe, is measured in units of (g/m^2^), with AGB specifically referring to the total organic matter of vegetation above the ground surface. In a broader context, the aboveground components of vegetation, including branches, leaves, and fruits, are also recognized as constituents of biomass at a given moment. Furthermore, the aboveground biomass of deceased trees is also factored into biomass assessments [5,6].

Despite its significance, traditional AGB monitoring methods face numerous challenges and limitations. These conventional approaches often involve destructive operations on crops, such as cutting and harvesting in specific areas, to measure fresh or dry weight based on different varieties [7,8]. However, these methods are time consuming, labor intensive, and possess limited coverage. They are further constrained by factors such as topography and land use. Additionally, the sampling process typically covers only a small fraction of experimental plots, potentially resulting in estimation biases for non-uniform plots.

With the continuous advancement of machine learning and deep learning algorithms, non-destructive methods are progressively replacing conventional monitoring techniques. Morais [9] and colleagues conducted a comprehensive examination of 26 literature sources, focusing on the application of machine learning and remote sensing data in predicting AGB in grasslands. Their findings revealed limitations in the universality and model validation of machine learning algorithms. In a different approach, Revenga [10] and Revenga’s team integrated machine learning techniques with high-resolution LiDAR data, utilizing an extremely randomized tree regression model for real-time AGB prediction. They achieved a noteworthy predictive performance with an R^2^ value of 0.48 at a spatial resolution of 0.35 m^2^. Gao [11] and collaborators employed airborne LiDAR and hyperspectral remote sensing data, extracting crucial features from both data types. Their optimized AGB models, constructed using random forests and multi-step regression methods, exhibited high accuracy levels for various tree species. Furthermore, Zhang [12] and Zhang’s team estimated maize biomass under different nitrogen fertilizer levels in Pingshan County, Jilin Province, utilizing low-altitude unmanned aerial vehicles and hyperspectral images. Employing the XGBoost model, they achieved high-precision predictions for fresh weight and dry weight AGB, particularly during the V6 growth stage (R^2^ = 0.81, RMSE = 0.27 t/ha).

However, the integration of machine learning into AGB prediction methods often requires manual intervention. The manual extraction and selection of crop phenotypic or physiological parameters as features from source data introduce subjectivity and inconsistency, influenced by the operator’s experience. Further feature selection requires specialized knowledge and subjective judgment, potentially leading to result uncertainties.

Compared to traditional machine learning, deep learning exhibits significant advantages in biomass prediction [13,14]. Firstly, deep learning constructs end-to-end networks that can directly learn and forecast AGB from sensor data, eliminating the need for manual feature extraction. This reduces reliance on domain-specific knowledge, enhancing system intelligence and real-time capabilities. Schreiber et al. [15] introduced an approach using visible spectrum images captured by drones, artificial neural networks, and convolutional neural networks for estimating the AGB of Brazilian wheat. This method bypasses feature extraction by directly inputting wheat images into the model to obtain predictions. Buxbaum et al. [16] proposed a deep learning method for directly predicting the biomass of lettuce plants from color and depth image data, particularly suitable for growth conditions involving self-occlusion or neighboring plant obstruction. This method demonstrated outstanding performance on a large-scale test dataset, significantly outperforming previous approaches.

Furthermore, deep learning models possess the capability to automatically learn complex relationships and patterns within large-scale and diverse datasets, providing a more robust processing capacity and comprehensive capture of the intricacies of crop growth. Additionally, they exhibit increased efficiency in handling large datasets, thereby enhancing prediction accuracy and generalization. For instance, Ma et al. [17] proposed a straightforward method for estimating the AGB of winter wheat using on-site digital images and a deep convolutional neural network (DCNN). Despite various influencing factors, the DCNN demonstrated optimal robustness with a high coefficient of determination (R^2^ = 0.808) and low root mean square error (RMSE = 0.8913 kg/plot, NRMSE = 24.95%). Similarly, Castro et al. [18] introduced and evaluated a method based on deep learning and RGB images captured by unmanned aerial vehicles (UAVs). They examined two convolutional neural network (CNN) models, namely AlexNet and ResNet18, and compared them with the previously used VGGNet in grass biomass estimation. The results indicated that AlexNet and ResNet18 achieved commendable performance, promising enhanced efficiency in estimating grass biomass.

### 1.1. Related Work

#### 1.1.1. Biomass Prediction Based on Images

RGB images are captured by conventional optical digital cameras, which offer the advantages of high resolution and low cost. UAVs possess advantages such as high spatial resolution, flexible perspectives, and the ability to access multiple locations in a short period. Leveraging these characteristics, some researchers have utilized images captured by UAVs to estimate the AGB [19,20,21,22,23]. This integrated use of digital cameras and UAVs provides an effective and cost-efficient approach for accurately estimating the AGB. In addition to directly predicting biomass values, there are studies that use image data to predict the relative content of a specific plant or crop, such as the percentage occupied, among other metrics [24,25,26]. Most of these studies analyze different grass contents in grasslands, for instance, Skovsen [27] and colleagues utilized convolutional neural networks to determine the biomass species composition of mixed crops from high-resolution color images. Data collection was conducted at four experimental sites over three growing seasons, and the method excelled in predicting the relative biomass content of clover (R^2^ = 0.91). Narayanan [28] and Narayanan’s team employed weakly supervised multi-objective deep learning methods to extract grass phenotypes and biomass percentages from a small dataset.

In contrast to RGB image information, multispectral and hyperspectral images often carry richer spectral and band information, albeit at a higher cost and lower cost-effectiveness. Some researchers have utilized hyperspectral images for biomass prediction. For instance, Li [29] and colleagues employed a low-altitude UAV to acquire RGB and hyperspectral image data of potato canopies at two growth stages, estimating AGB and predicting crop yield. They proposed a Partial Least Squares Regression model based on the entire wavelength spectrum, demonstrating improved yield prediction accuracy (R^2^ = 0.81). Liu [30] and team explored various machine learning methods, including a Support Vector Machine (SVM), Random Forest (RF), and Gaussian Process Regression (GPR), to extract multiple variables (including the Canopy Original Spectrum (COS), First Derivative Spectrum (FDS), Vegetation Index (VI), and Crop Height (CH)) from UAV hyperspectral images for estimating potato AGB. In the realm of multispectral image information, some scholars have combined machine learning or deep learning methods to predict crop biomass [31,32,33].

#### 1.1.2. Contrastive Learning

Contrastive learning, recognized as an effective method for representation learning, has garnered significant achievements across various domains, drawing widespread attention. The foundation of contrastive learning was laid by the earliest self-supervised learning proposed by Becker and Hinton [34], which emphasized maximizing consistency between similar inputs and underscored the intrinsic relationships among samples. Representative methods in contrastive learning include SimCLR [35] and MoCo [36], both defining positive samples as augmented samples from the same input data without the need for labels. By maximizing the feature similarity of similar samples, these methods achieve clustering of similar samples in the feature space. Supervised contrastive learning approaches, such as SupCon [37], define positive samples as those belonging to the same category. This method trains by emphasizing the similarity among samples of the same category, providing effective representations for classification tasks. The aforementioned approaches have played a pivotal role in advancing contrastive learning methodologies and contributing to their widespread application and recognition.

In the context of deep regression, the application of contrastive learning methods is referred to as deep regression. Wang et al. [38] introduced a novel approach known as CRGA, designed for the unsupervised extension of visual estimation in target domains. CRGA integrates contrastive domain generalization and contrastive self-training adaptation, effectively narrowing the features of similar gaze directions and achieving robust regression outcomes. Barbano et al. [39] proposed a new contrastive learning regression loss for accurately predicting brain age through MRI scans. This method exhibits outstanding performance in handling diversity in datasets from different sites and scanners, demonstrating optimal results in the OpenBHB challenge. Zha et al. [40] introduced Rank-N-Contrast (RNC), a framework for learning continuous representations in regression. In contrast to traditional deep regression models, RNC emphasizes the ranking of contrastive samples in the target space, ensuring that the learned representations align with the target order. Theoretical and empirical results suggest that RNC not only outperforms in performance but also demonstrates significantly improved robustness, efficiency, and generalization capabilities. Keramati et al. [41] presented ConR, a contrastive regularization method, to address imbalanced regression problems in deep learning. Through contrastive regularization, ConR simulates the similarity of global and local labels in feature space, preventing the merging of features from minority-class samples into majority-class samples, and effectively resolves imbalanced regression issues in deep learning.

### 1.2. Main Contributions

While there have been considerable advancements in biomass prediction methodologies utilizing image data and deep learning techniques, the current body of literature exhibits a noticeable oversight regarding the intricate relationship between image features and corresponding labels. Moreover, prevalent methodologies predominantly rely on classical convolutional neural networks (CNNs) or their refined iterations, often overlooking a thorough examination of the intricate relationships inherent in deep regression tasks involving images and labels. Acknowledging these significant gaps, this study conducts a comprehensive investigation into the nuanced relationships between image features and labels within the realm of deep regression tasks. To address these identified deficiencies, we propose an augmented Supervised Contrastive Deep Regression (SCDR) framework specifically tailored for biomass prediction. Additionally, we introduce a novel biomass prediction network optimized for image data, termed the U-Like Hierarchical Residual Fusion Network (BioUMixer).

**Figure 1:**
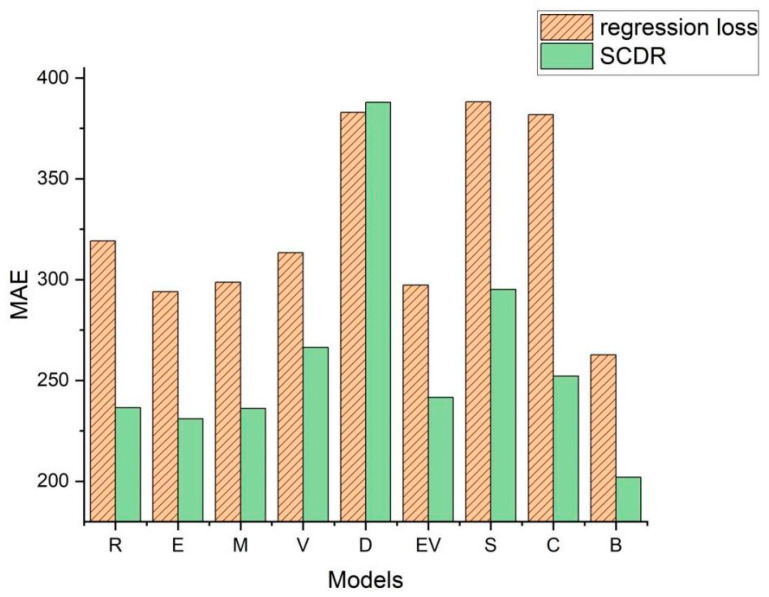
Displays the Mean Absolute Error (*MAE*) values for various models under two distinct loss functions. The corresponding letters and models are as follows: R—ResNet50; E—EfficientNetV2; M—MLP_Mixer; V—ViT; D—DaViT; EV—EfficientViT; S—SwinTransformers; C—ConvNeXtV2; and B—BioUMixer.

The essence of biomass prediction via deep learning resides in the intricate endeavor of accurately projecting continuous labels employing deep learning methodologies. Given the limited consideration accorded to image and label features in existing biomass prediction frameworks, this paper introduces a paradigm shift towards supervised contrastive learning within the domain of deep regression, termed SCDR. Inspired by established methodologies such as InfoNCE [42] and ConR [41], SCDR embodies a comprehensive approach, both algorithmically and structurally, aligning more closely with the demands and underlying motivations inherent in deep regression tasks. As a result, SCDR not only addresses the aforementioned limitations but also significantly enhances the efficacy of downstream deep regression tasks, including the prediction of Above-Ground Biomass (AGB). Figure 1 illustrates the Mean Absolute Error (*MAE*) values of various models on the Pepper_Biomass dataset under both conventional regression loss and the SCDR method.

Our primary contributions are as follows:

1. This paper produces the Pepper_Biomass dataset, an exhaustive and carefully curated resource for pepper biomass research. The dataset consists of over 600 raw images, each carefully labeled with the appropriate biomass tag, attesting to the rigorous organization process and high-quality standards of the dataset.
2. An enhanced SCDR was introduced for deep regression tasks. This approach was meticulously tailored to align more closely with the requirements and motivations inherent in such tasks, ultimately bolstering the performance of downstream deep regression tasks, notably in biomass prediction.
3. The U-Like Hierarchical Residual Fusion Network (BioUMixer) was introduced as a dedicated network designed for image data. BioUMixer undertakes feature extraction on biomass image information, fostering robust information exchange and fusion across modules. This facilitates the harmonious intertwining of global and local information features within the images.

## 2. Materials and Methods

### 2.1. SCDR—Supervised Contrastive Learning for Deep Regression

**Figure 2:**
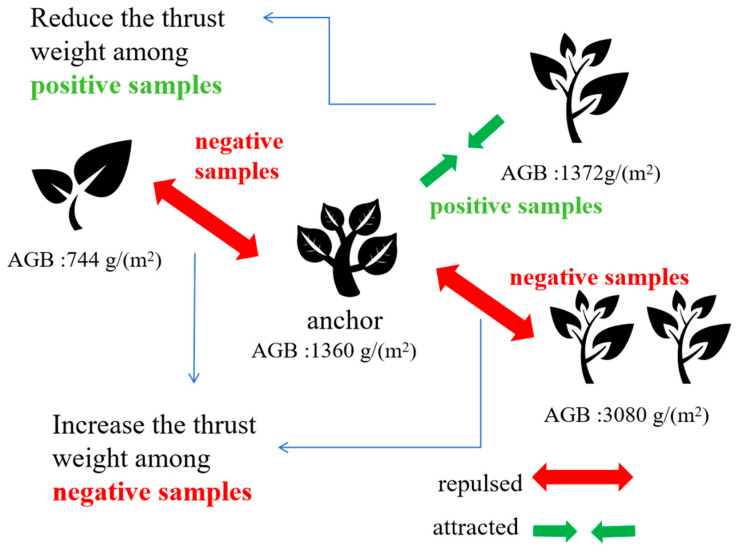
Schematic diagram of thrust calculation between sample pairs.

The fundamental innovation of SCDR resides in its unique methodology for computing contrastive loss, which holds pivotal significance in mitigating the regression challenges inherent in biomass prediction tasks utilizing deep learning methodologies. This pioneering framework encompasses two critical components: Firstly, the utilization of advanced techniques such as data augmentation and anchor data selection to generate two augmented samples for each data point, thereby enriching the training dataset and bolstering model robustness. Subsequently, SCDR integrates supervised contrastive learning with thrust weight computation, wherein weight calculation simultaneously considers both sample features and label similarities. This integration ensures that samples with analogous biomass values are strategically positioned closer in the feature space, facilitating precise prediction, while those with substantial biomass disparities are delineated by greater distances, thereby augmenting the model’s discriminative capabilities. Notably, in calculating thrust between negative sample pairs, the framework meticulously accounts for both label values and the similarity of feature pairs for negative samples, thus mitigating interference from inaccurately labeled pairs and further amplifying the accuracy and comprehensiveness of thrust calculations. This comprehensive approach, amalgamating both types of information, not only advances the accuracy of biomass prediction but also furnishes an extensive comprehension of the interplay between image features and biomass labels, as exemplified in the schematic depiction in Figure 2, illustrating thrust computation between sample pairs.

#### 2.1.1. Problem Definition

Given a dataset containing N samples, denoted as ${\left\{(x_{i},y_{i})\right\}}_{i=0}^{N}$, where $x_{i}\in R^{d}$ is the input sample data, $y_{i}\in R^{d^{\prime }}$ is the value of the label corresponding to the input sample. d and $d^{\prime }$ are the dimensions of the representation space to which the input samples and their corresponding labels belong. Given a feature encoder $\varepsilon \left(\cdot \right)$ and a regression prediction function $R\left(\cdot \right)$, these two components form a regression model. The goal is to train the regression model $R\left(\varepsilon \left(\cdot \right)\right)$ such that the final model output ${\hat{y}}_{i}=R\left(\varepsilon \left(x_{i}\right)\right)$ minimizes the L1 distance from the true label value $y_{i}$, given the sample dataset. In this section, $x_{i}$ is the crop image data, $y_{i}$ is the real AGB value corresponding to $x_{i}$, and ${\hat{y}}_{i}$ is the AGB value predicted by the regression model.

#### 2.1.2. Anchor Data and Pair Selection

For the input data $x_{i}$, we perform a generalization operation based on the modal type of the data to produce two generalized samples. For a given dataset ${\left\{(x_{i},y_{i})\right\}}_{i=0}^{N}$, the set of samples after generalization is ${\left\{({\tilde{x}}_{j},{\tilde{y}}_{j})\right\}}_{j=0}^{2N}$. Where ${\tilde{x}}_{2k}=t\left(x_{k}\right)$, ${\tilde{x}}_{2k-1}=t^{\prime }\left(x_{k}\right)$, and $t\left(\cdot \right)$ and $t^{\prime }\left(\cdot \right)$ are independently sampled generalization operations. That is, for each sample $x_{k}$, the samples after generalization are ${\tilde{x}}_{2k}$ and ${\tilde{x}}_{2k-1}$, where k∈[0,N]. And for the label of the corresponding data of the expanded samples, there is ${\tilde{y}}_{2k}={\tilde{y}}_{2k-1}=y_{k}$, i.e., the label value of the expanded data does not change. SCDR plays a synergistic role in the selection of sample pairs and in changing the distance between sample pairs. For each sample after augmentation, SCDR first determines whether the samples are paired with each other, and, if they are paired, whether they are positive sample pairs or negative sample pairs. After that, in the case of at least one negative pair, the sample is considered anchor data and SCDR pulls the positive pairs together and rejects the negative pairs relatively.

(1) Pairing Choice

**Figure 3:**
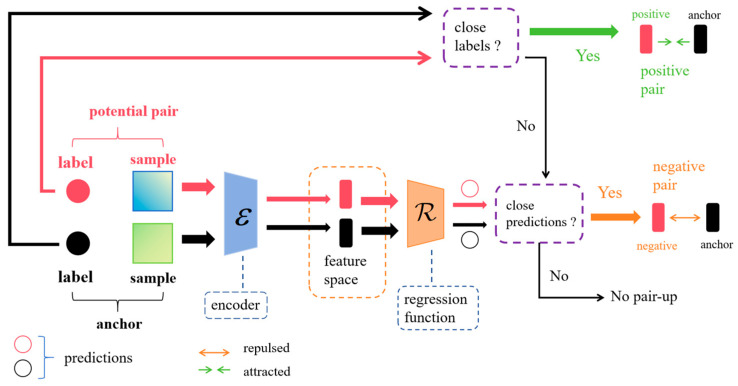
Pairwise selection process of SCDR. For every augmented sample pair, SCDR initially evaluates the proximity of their labels; in the event of close proximity, the pair is categorized as a positive sample pair. Concurrently, the samples are encoded and subjected to a regression function to derive predicted values. Should the labels of a sample pair not exhibit proximity in distance, yet their predicted values demonstrate closeness, they are classified as negative sample pairs. Any cases beyond the aforementioned criteria are not paired.

Consider two samples in the augmented sample set, $\left({\tilde{x}}_{i},{\tilde{y}}_{i}\right)$ and $\left({\tilde{x}}_{j},{\tilde{y}}_{j}\right)$, which are passed through the feature encoder $\varepsilon \left(\cdot \right)$ to produce two feature vectors $v_{i}$ and $v_{j}$, i.e., $v_{i}=\varepsilon \left(x_{i}\right)$ and $v_{j}=\varepsilon \left(x_{j}\right)$, respectively. After that, the regression function $R\left(\cdot \right)$ is used to obtain the predicted values ${\hat{y}}_{i}$ and ${\hat{y}}_{j}$, respectively. The predicted and actual labeled values are used to determine whether the examples should be positive sample pairs, negative sample pairs, or unpaired. SCDR determines the similarity between labeled or predicted values in the augmented samples by defining a similarity threshold at ω. Given a similarity function, $Sim\left(\cdot ,\cdot \right)\in R$, two labeled values ${\tilde{y}}_{i}$ and ${\tilde{y}}_{j}$ (or predicted values ${\hat{y}}_{i}$ and ${\hat{y}}_{j}$) are considered similar if they have $Sim\left({\tilde{y}}_{i},{\tilde{y}}_{j}\right)\geq \omega$ (or $Sim\left({\hat{y}}_{i},{\hat{y}}_{j}\right)\geq \omega$). Two samples are considered positive if they have similar labeled values, and negative if they do not have similar labeled values but have similar predicted values. Outside of these two cases they are considered unpaired. The detailed process of pairwise selection for SCDR is shown in Figure 3.

(2) Selected Anchor Data

For each sample *j*, $\left({\tilde{x}}_{j},{\tilde{y}}_{j}\right)$, we denote the set of feature vectors for positive and negative sample pairs for that example as $P_{j}^{+}={\left\{(v_{p})\right\}}_{p=0}^{N_{j}^{+}}$ and $P_{j}^{-}={\left\{(v_{q})\right\}}_{q=0}^{N_{j}^{-}}$, respectively, where $N_{j}^{+}$ is the number of positive samples and $N_{j}^{-}$ is the number of negative samples. For sample *j*, sample $\left({\tilde{x}}_{j},{\tilde{y}}_{j}\right)$ is considered as anchor data if $N_{j}^{-}>0$ is available.

#### 2.1.3. Supervised Contrastive Loss for Deep Regression

For the SCDR method, we introduce the loss function $L_{SCDR_{j}}$. For sample *j*, if it is not selected as anchor data, there is $L_{SCDR_{j}}=0$. Otherwise, this loss function draws the positive sample pairs closer in the feature space, and at the same time draws the negative sample pairs closer to each other proportionally according to the similarity of their labels and eigenvalues, as shown in the following formula:

(1) $$L_{SCDR_{j}}=-\log\frac{1}{N_{j}^{+}}\sum_{v_{i}\in P_{j}^{+}}\frac{\exp(v_{j}\cdot v_{i}/\tau )}{\sum_{v_{p}\in P_{j}^{+}}\exp(v_{j}\cdot v_{p}/\tau )+\sum_{v_{q}\in P_{j}^{-}}S_{j,q}\exp(v_{j}\cdot v_{q}/\tau )}$$

where τ is the temperature parameter and $v_{j}$ is the feature vector generated by the anchor data after the feature encoder. $S_{j,q}$ is the thrust between each negative sample pair, which is given by

(2) $$S_{j,q}=f_{S}\left(\eta_{j},\;Sim_{l}\left({\tilde{y}}_{j},{\tilde{y}}_{q}\right),\;Sim_{f}\left(v_{j},v_{q}\right)\right)$$

In Equation (2), $f_{S}\left(\cdot \right)$ is the negative sample pair pushover calculation function, ${\tilde{y}}_{q}$ is the label value of sample $x_{q}$, $\eta_{j}$ is the weight coefficient, and $Sim_{l}\left(\cdot ,\cdot \right)$ and $Sim_{f}\left(\cdot ,\cdot \right)$ are the label similarity calculation function and sample feature similarity calculation function, respectively. $S_{j,q}$ the computed value of $\eta_{j}$ is proportional to and inversely proportional to $Sim_{l}\left({\tilde{y}}_{j},{\tilde{y}}_{q}\right)$ and $Sim_{f}\left(v_{j},v_{q}\right)$. We consider both the similarity between labeled values and the similarity of negative sample pair features when calculating the thrust between negative sample pairs. Considering both kinds of information simultaneously makes the thrust calculation more accurate and comprehensive, and also reduces the interference of wrong labels on the thrust calculation.

In Equation (2), the absence of weight calculation gives rise to notable issues. Firstly, the model may inadequately address crucial yet infrequently encountered negative samples, resulting in diminished efficacy in modeling these instances. Secondly, with prevailing negative samples, undue model concentration may induce overfitting of their relationships, thereby adversely affecting overall generalization performance.

The incorporation of weight calculation substantially enhances the capacity of SCDR to accurately evaluate relationships between samples. This augmentation allows for a more focused consideration of significant negative samples, effectively mitigating concerns related to overfitting concerning commonly occurring negative samples. Fine-tuning of weights enables optimization of the model’s learning process across diverse samples, facilitating more adept adaptation to intricate relationships inherent in real-world scenarios. Consequently, this augmentation significantly elevates the overall performance of the SCDR method in the realm of deep regression tasks.

Therefore, incorporating weight calculation into the SCDR method not only rectifies potential issues arising from the absence of such consideration but also bestows upon the model a heightened capacity for precise and reliable relationship modeling. This refinement empowers the model to manifest superior performance, particularly when confronted with intricate datasets of a complex nature.

The loss function $L_{SCDR}$ is the average of the loss summed over all the generalized samples, i.e.,

(3) $$L_{SCDR}=\frac{1}{2N}\sum_{j=0}^{2N}L_{SCDR_{j}}$$

Finally, the total loss function for deep regression for supervised comparisons is a weighted sum of $L_{SCDR}$ and regression losses:

(4) $$L_{sum}=\alpha L_{R}+\beta L_{SCDR}$$

Through the above SCDR method and loss function, from the view of the sample as a whole, we make the distance between the model predicted values and labeled values decrease; at the same time, for the inner sample, through the selection of the positive and negative sample pairs and the change in their relative distances, we make the samples themselves and the labeled values in the feature space in accordance with their relative order, which further increases the prediction accuracy.

### 2.2. BioUMixer—U-Like Hierarchical Residual Fusion Network

In preceding investigations concerning image-based biomass prediction, the prevalent utilization of comparatively straightforward models has been observed. These inquiries frequently employ Convolutional Neural Network (CNN) models initially designed for classification tasks directly in the context of biomass prediction, lacking a comprehensive consideration of the distinct attributes characterizing biomass prediction tasks. It is imperative to underscore that the essence of biomass prediction tasks lies in the precise estimation of continuous and variable biomass values, as opposed to the mere classification of static images.

The distinctiveness intrinsic to biomass prediction becomes evident in the imperative to quantify the volume of living organisms. This necessitates models endowed with heightened precision and regression capabilities to apprehend the intricacies of biological structures and tissue information encapsulated within images. In contrast, traditional classification tasks are inclined to prioritize the delineation of object categories over specific quantitative values. Thus, when confronting biomass prediction tasks, models must cultivate a profound understanding of the spatial distribution, morphological features, and quantitative relationships governing biological entities within images. A meticulous consideration of the idiosyncrasies inherent in biomass prediction tasks is essential to efficaciously address this challenge.

In the developmental and training phases of models, the imperative arises for the incorporation of more adaptive and intricate deep learning architectures. This is requisite to effectively encapsulate biological information within images and to ensure the precise and continuous estimation of biomass. This specialized deep learning paradigm tailored for biomass prediction tasks inherently surpasses the efficacy of simplistic classification models, adeptly catering to the unique prerequisites of precision and granularity entailed in biomass prediction.

**Figure 4:**
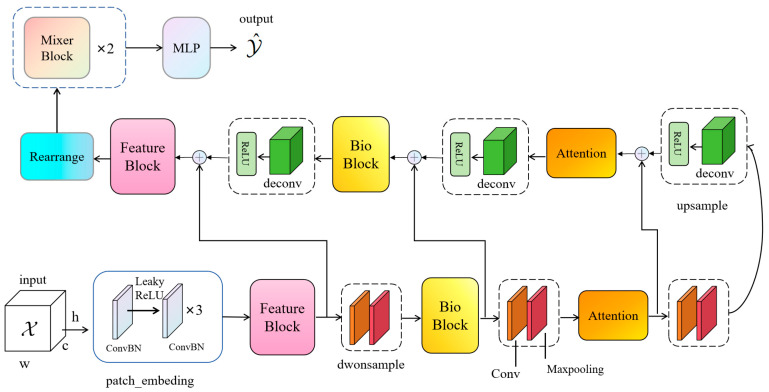
Overall architecture of BioUMixer. The input is introduced into the network via patch embedding. As it traverses each feature extraction module (FeatureBlock, BioBlock, Attention), shortcut branches are elongated to establish connections between the input and the subsequent module of identical nomenclature. Ultimately, the feature maps undergo processing through a MixerBlock and MLP to culminate in the ultimate output.

To surmount this challenge, this paper posits the introduction of a novel network christened BioUMixer, featuring a U-like hierarchical residual fusion structure. This network seamlessly integrates BioBlock and FeatureBlock for meticulous feature extraction from biomass image information. Additionally, the incorporation of the SimAM lightweight attention mechanism expeditiously facilitates attention weight calculations by resolving the energy function, thereby extracting pivotal information from images without incurring significant computational overhead. Lastly, the U-like hierarchical residual structure amplifies information exchange and fusion among modules, effectuating the symbiotic integration of global and local information features within images. The comprehensive architectural schematic of BioUMixer is delineated in Figure 4.

#### Deep Feature Extraction Module

(1) FeatureBlock

**Figure 5:**
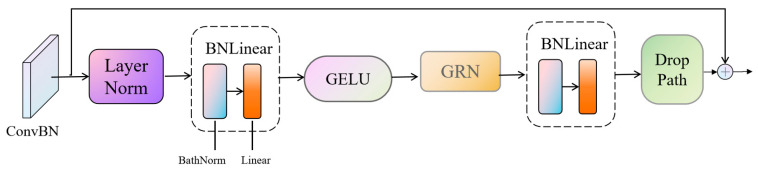
Flowchart of FeatureBlock structure.

The FeatureBlock, integrating ConvNeXt and Transformer structures, is employed to enhance the model’s perceptual range in scenarios characterized by dense vegetation and complex background images. The introduction of BatchNorm serves to stabilize the feature distribution, thereby augmenting the model’s robustness. The utilization of Global Response Normalization (GRN) systematically addresses the challenge of feature folding, ensuring the comprehensive capture of global information within the images [43]. Concurrently, the integration of DropPath as a regularization technique effectively mitigates overfitting, consequently contributing to the enhanced accuracy of biomass prediction. The architecture of FeatureBlock is illustrated in Figure 5.

(2) BioBlock

**Figure 6:**
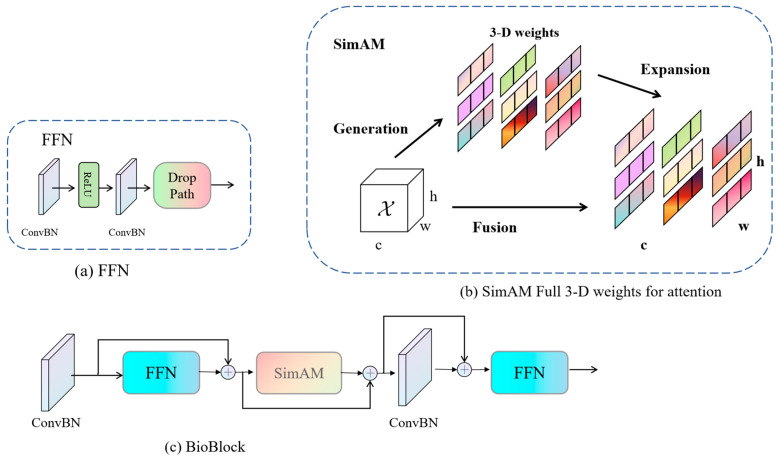
Overall structure of BioBlock: (**a**) FFN module structure; (**b**) SimAM, a full three-dimensional weighted attention. In each subfigure, consistent colors indicate the application of a single scalar to each channel, spatial position, or feature point; (**c**) BioBlock structure.

The objective of the BioBlock design is to intricately merge primary and deep features through residual connections, enabling a highly detailed extraction of image features. The incorporation of a memory-efficient “sandwich layout” design and a parameter reallocation strategy not only contributes to an improved model efficiency but also renders it more apt for the processing of large-scale biomass image data [44]. The introduction of the SimAM attention mechanism, amalgamating channel, and spatial attention facilitates more precise 3D-weight calculations, allowing for a more accurate capture of features in different regions of the image [45]. Consequently, this enhancement effectively improves the accuracy of biomass prediction. Figure 6 presents the schematic representation of BioBlock’s structure, offering a clear depiction of its intricate information extraction process within the context of biomass prediction tasks.

(3) U-Like Hierarchical Residual

**Figure 7:**
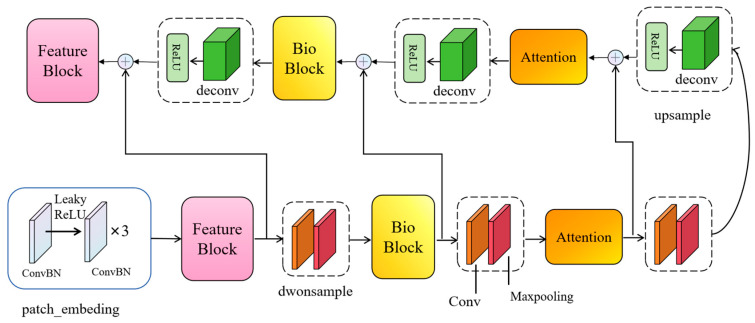
Depicts the U-Like hierarchical residual connections. The structural framework, as depicted in Figure 4, embodies a U-shaped network configuration.

Unet-type networks have exhibited substantial advantages in the domain of image segmentation owing to their encoder-decoder architecture and the incorporation of skip connections [46,47,48,49,50]. The U-like hierarchical residual connections proposed in this paper extend the Unet network structure and adeptly integrate features of diverse scales through skip connections. This architectural framework demonstrates exceptional efficacy in biomass prediction tasks, adeptly harmonizing global and local information within images, thereby underscoring its significance in extracting nuanced features from biomass images. This design accentuates the distinctive advantages it holds within the realm of biomass prediction.The overall structure of the U-Like Hierarchical Residual is illustrated in Figure 7.

### 2.3. Experimental Setup

Baseline models:

In tasks involving the deep learning prediction of biomass, ResNet [51] is a commonly employed network. As there is limited research specifically dedicated to proposing networks tailored for biomass prediction tasks, we chose networks that have demonstrated superior performance in visual classification tasks, including ResNet50, ConvNeXtV2 [43], EfficientViT [44], EfficientNetV2 [52], ViT [53], DaViT [54], SwinTransformer [55], and MLP_Mixer [56].

Evaluation Metrics:

To assess the performance of the proposed methods and models, we utilized three evaluation metrics: Root Mean Square Error (*RMSE*), Mean Absolute Error (*MAE*), and Mean Absolute Percentage Error (*MAPE*). RMSE serves as a standard deviation measure for predicting errors. The computation involves squaring the prediction errors of each observed value, calculating the mean, and then taking the square root. It is notable for being highly influenced by outliers, as the squared errors amplify the impact of outliers. RMSE is more sensitive to large errors, making it suitable for scenarios where sensitivity to errors is crucial. The mathematical formula is given by

(5) $$RMSE=\sqrt{\frac{1}{n}\sum_{i=1}^{n}{\left(y_{i}-{\hat{y}}_{i}\right)}^{2}}$$

The *MAE* (Mean Absolute Error) is the average absolute value of prediction errors. The calculation involves taking the absolute value of the prediction error for each observed value and computing the mean.

(6) $$MAE=\frac{1}{n}\sum_{i=0}^{n}\left|y_{i}-{\hat{y}}_{i}\right|$$

The *MAPE* (Mean Absolute Percentage Error) represents the average absolute error in percentage form. The calculation involves taking the absolute value of the prediction error for each observed value and then dividing it by the actual observed value.

(7) $$MAPE=\frac{1}{n}\sum_{i=0}^{n}\left|\frac{y_{i}-{\hat{y}}_{i}}{y_{i}}\right|$$

Implementation details:

This experiment was conducted on the Ubuntu 20.04.4 LTS 64 operating system (Canonical Ltd., London, UK), utilizing an Intel^®^ Xeon^®^ Silver 4214 processor with a clock speed of 2.20 GHz and 32 GB of RAM (Intel, Santa Clara, CA, USA). The graphics processing unit (GPU) employed was an NVIDIA Tesla T4 with 16 GB of video memory (Nvidia, Santa Clara, CA, USA). We utilized the Adam optimizer, performed training for 80 epochs, and implemented cosine annealing to decay the learning rate.

## 3. Experiments

### 3.1. Dataset

In September 2023, we conducted the inaugural data collection for pepper biomass in Shawan City, Xinjiang. Twenty one-meter × three-meter subplots were delineated using one-meter-high PVC plastic stakes and plastic lines arranged in a serpentine pattern, each subplot accompanied by a numbered placard. Utilizing drone aerial imagery ensured both the consistency and comprehensiveness of the gathered data.

**Figure 8:**
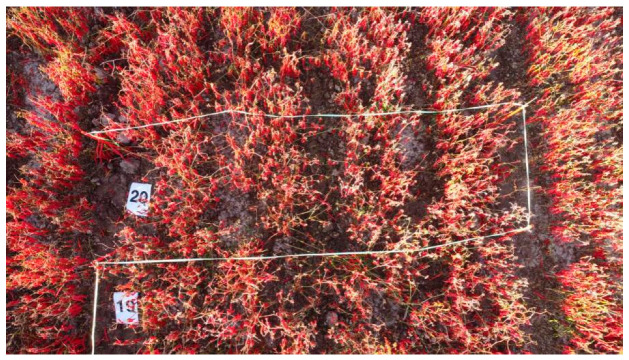
An example of chili peppers captured through aerial photography using a drone.

In October 2023, data collection for bell pepper biomass was carried out in Yanqi County, Xinjiang. A total of 80 1-m × 1.5-m subplots, each covering a row of bell pepper plants, were demarcated using PVC pipes. Employing two sets of parallel measurements and handheld selfie sticks equipped with smartphones facilitated the acquisition of image data. Trained harvesters, wearing gloves, harvested the bell peppers, which were then individually labeled with corresponding numbers, weighed, and recorded. Additionally, during both harvest sessions, above-ground stems exceeding 30 cm in length were harvested, with the total mass encompassing both fruit and stem weights. In response to post-captured images, we employed various image enhancement techniques, encompassing cropping, flipping, adjusting color contrast, and normalization. This enhancement process bolstered the robustness and diversity of the dataset. Additionally, images that could not be recognized were systematically excluded. The chili pepper image data collected in September and October were amalgamated and collectively referred to as “Pepper_Biomass”. A sample image of the chili pepper data is shown in Figure 8. Additional details concerning the data collection process are elaborated upon in Table 1. For a comprehensive overview of the specifications pertaining to the capturing devices, we direct the reader’s attention to Table 2.

**Figure 9:**
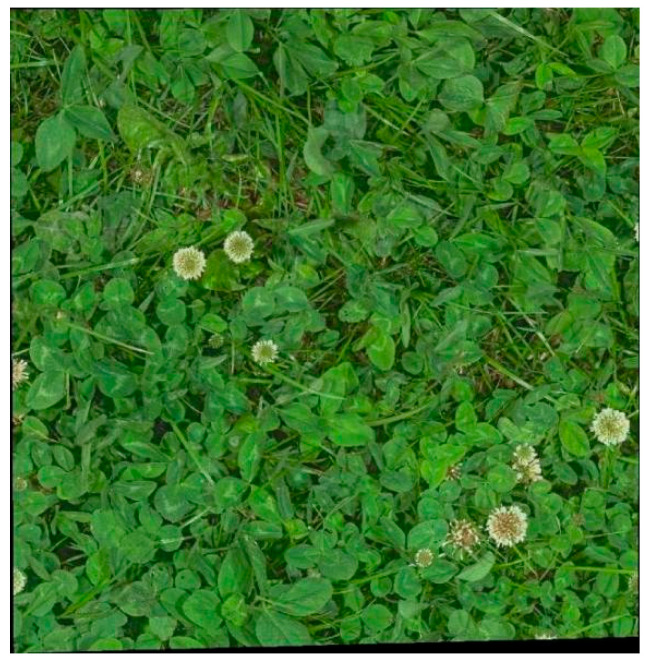
Sample GrassClover dataset.

GrassClover is a diverse open dataset compiled from outdoor agricultural environments, encompassing a variety of images and biomass data. The images feature dense mixtures of grass and clover communities, characterized by significant occlusion and the presence of weeds. The dataset’s primary challenge lies in predicting the species composition within the vegetation images and biomass, crucial for understanding the impact of local species composition on mixed-crop fertilization and treatments. The dataset was collected using three different acquisition systems, with a ground sampling distance ranging from 4 to 8 pixels/millimeter. The observed mixed crops exhibit variations in settings (field vs. plot experiments), seed composition, yield, establishment years, and seasonal timing. Among the dataset images, 435 are designated for biomass prediction.An example image from the GrassClover dataset is shown in Figure 9.

The datasets Pepper_Biomass and GrassClover were partitioned into training and testing sets. Subsequently, exclusive data augmentation was applied solely to the training set. Following augmentation, Pepper_Biomass and GrassClover comprised approximately 2000 and 1000 images, respectively.

### 3.2. Experimental Results

In this investigation, three regression-oriented loss functions—namely, Focal_L1 [57], RNC, and ConR—were meticulously chosen for a comparative analysis. The performance results of nine models, including our novel proposition, BioUMixer, on the Pepper_Biomass dataset, are comprehensively presented in Table 3. Notably, SCDR emerges as the preeminent loss function, consistently surpassing Focal_L1 and the other two alternatives in predicting Pepper_Biomass across a majority of the models. This observation implies that SCDR effectively adjusts sample distances by evaluating label and feature similarities, thereby mitigating potential biases that may arise with Focal_L1. Specifically, SCDR_Loss demonstrates a significant average decrease of 4.68% in *MAPE* across all models when compared to Focal_L1, indicating a substantial enhancement in overall model performance.

BioUMixer exhibits commendable performance exclusively with the Focal_L1 loss function, showcasing *RMSE*, *MAE*, and *MAPE* values of 316.03, 262.66, and 0.136, respectively. Its efficacy lies in meticulous feature extraction from images, achieving superior biomass prediction by seamlessly integrating global and local features through multi-layer residual structures. Upon incorporating the proposed SCDR method, BioUMixer attains optimal performance, yielding *RMSE*, *MAE*, and *MAPE* values of 252.18, 201.98, and 0.107, respectively. Remarkably, models relying on pure Transformer structures manifest mediocre performance in biomass prediction tasks employing image data. This may be attributed to limitations in capturing local information, susceptibility to overfitting, and inadequate modeling of translation invariance.

Although CNN-type and hybrid models surpass pure Transformers, they have not yet attained optimal levels of performance. CNNs excel in capturing image information but exhibit relative weakness in fusing global and local data, particularly in the context of biomass prediction tasks. The proposed BioUMixer addresses this by introducing a U-like hierarchical residual structure, thereby enhancing information exchange and fusion between modules to facilitate improved convergence of global and local information in images—attributes particularly advantageous for biomass prediction tasks.

Parallel experiments conducted on the GrassClover dataset, involving nine models and four loss functions, consistently underscore the effectiveness of the proposed SCDR method. When coupled with SCDR, BioUMixer achieves peak performance on GrassClover, exhibiting *RMSE*, *MAE*, and *MAPE* values of 47.92, 31.74, and 0.192, respectively.The specific results are presented in Table 4.

**Figure 10:**
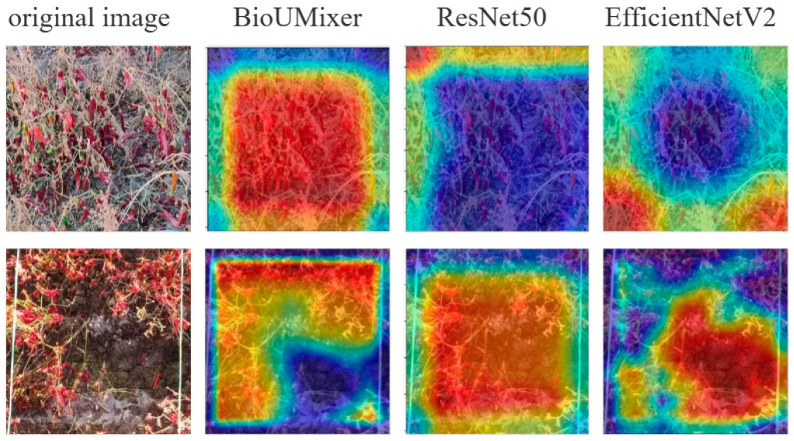
Attention and region of interest visualization.

Figure 10 presents attention visualization images of the top three models ranked by evaluation metrics on the Pepper_Biomass dataset. The first column displays the original image data fed into the models, while the subsequent three columns exhibit attention visualization images generated by the BioUMixer, ResNet50, and EfficientV2 models, respectively. Attention visualization images utilize a color mapping scheme to denote the degree of focus exerted by the models. Common color mappings include heatmap representations, where warmer colors such as red typically signify areas of high focus, while cooler colors such as blue or black denote areas of low attention.

From the visualizations, it is evident that the attention regions of the BioUMixer model closely align with densely populated regions of pepper fruits and stems in the original images, particularly noticeable in the second row of images. In the images of the second row, the lower-right corner corresponds noticeably to bare soil, while the peppers on the left and top sides form a right angle, aligning closely with the red regions in the heatmap. Conversely, the bare soil area in the lower right corner is represented by blue in the heatmap. This observation indicates that the BioUMixer model actively learns regions highly correlated with pepper biomass during its learning process. In contrast, attention regions in the heatmap of the other two models exhibit misalignment or excessive focus on areas unrelated to biomass contribution.

### 3.3. Ablation Study

(1) Module ablation experiments on BioUMixer

We conducted ablation experiments on key structural designs and modules of BioUMixer, systematically assessing their impact by replacing or removing them. Subsequently, we re-evaluated the model on two datasets under consistent conditions to validate the significance of our meticulously designed modules. Ablation focused on three critical aspects: U-Like Hierarchical Residual, Feature_block, and Bio_block. Five scenarios were tested: (1) only the main module network, excluding U-Like Hierarchical Residual, Feature_block, and Bio_block; (2) exclusively removing U-Like Hierarchical Residual; (3) solely removing Feature_block; (4) solely removing Bio_block; (5) the proposed BioUMixer model.The specific results are presented in Table 5.

The BioUMixer model, as proposed in this study, adeptly integrates the unique characteristics of the three modules. The observed performance enhancement resulting from the combination of these modules defies a simple additive relationship, affirming the optimal performance of the model and robustly validating the efficacy of the proposed modules.

(2) Parameter Ablation Experiment

In order to explore the effect of various parameters on the experimental results, we performed parameter ablation experiments on the Pepper_Biomass dataset. The experiments in Table 6 are the parameter ablation experiments for the coefficients β of the loss function of $L_{SCDR}$ in the total loss function, fixing α=1 (both α and β are weighting coefficients in Equation (4)). From Table 6, it can be seen that the performance of the model is optimized when β=3. Through comprehensive analysis, it becomes apparent that superior performance is achieved across all evaluation metrics when the weighting coefficient of SCDR_Loss surpasses that of the Focal_L1 regression loss. However, it is imperative to highlight that the weighting coefficient of SCDR_Loss is not boundless. Upon exceeding a threshold of three, a discernible decreasing trend in evaluation metrics is observed as the coefficient increases. This phenomenon can be attributed to the overarching imbalance in overall loss when the weighting coefficient of SCDR_Loss significantly surpasses that of the regression loss. Despite SCDR_Loss effectively incorporating the spatial relative order between positive and negative sample pairs and considering label distances in biomass prediction, substantial discrepancies in weighting coefficients may incline the model towards emphasizing the contrast between positive and negative sample pairs during training. This propensity has the potential to undermine the model’s proficiency in accurately predicting biomass based solely on image data.

In Table 7, τ is the temperature coefficients in Equation (1). When τ=0.2 the model is trained by SCDR and the best results are achieved on the biomass prediction task. In the realm of contrastive learning, the temperature hyperparameter assumes a pivotal role in governing the relative comparison between positive and negative samples. Lower temperatures bias the model towards prioritizing sample pairs characterized by heightened contrast, while higher temperatures foster a balance in importance between positive and negative samples, thereby facilitating a more comprehensive learning process. Within this framework, the temperature parameter serves as a modulating agent, finely tuning the sensitivity to contrast. In our investigation, we discerned that setting the temperature parameter τ to 0.2 yields optimal performance for the model. This observation suggests that, under this temperature setting, the model demonstrates an enhanced capacity to discern subtle disparities between positive and negative sample pairs, leading to a more proficient extraction of features and subsequent biomass prediction. Specifically, the lower temperature parameter (τ = 0.2) heightens the model’s sensitivity to sample pairs with pronounced contrast, thereby bolstering the precision and stability of biomass prediction. Furthermore, we noted a marginal decrease followed by a subsequent uptick in model performance within the temperature range of 0.3 to 1. This phenomenon may be ascribed to the inherent instability of the regression contrast system engendered by fluctuating temperature changes within this range. Conversely, as the temperature surpasses one, model performance experiences a downturn. This decline could be attributed to the heightened temperatures inducing an excessive balance in contrast, rendering it arduous for the model to discern nuanced disparities between positive and negative sample pairs, thereby compromising the accuracy of biomass prediction. It warrants emphasis that temperatures that are either excessively low or high prove unsuitable for biomass prediction tasks, thus underscoring the criticality of selecting the optimal temperature parameter.

**Figure 11:**
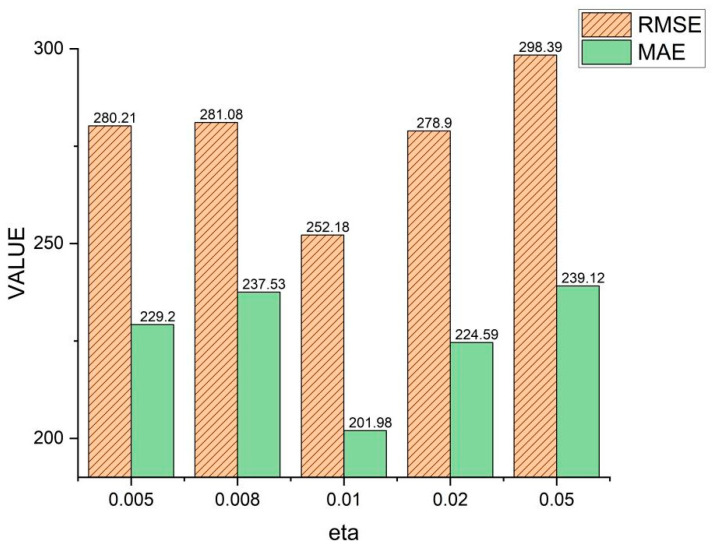
The ablation experiment of the parameter *η* (thrust calculation weight between SCDR sample pairs), where eta in the figure represents *η*.

Figure 11 shows the effect and influence of the SCDR samples on the η parameter in the thrust calculation and the model are optimized when η=0.01. When the value of η falls below 0.01, the model may disregard certain significant yet infrequently encountered negative samples, leading to a diminished capacity to model these instances effectively. Consequently, the model might fail to comprehensively capture specific patterns or relationships embedded within the dataset, thus adversely affecting its predictive efficacy. Moreover, excessively small values of η may prompt the model to excessively rely on a limited subset of samples, resulting in an incomplete comprehension of the overall data distribution and consequently hampering the model’s ability to generalize.

Conversely, as η surpasses the threshold of 0.01, the model may exhibit an undue emphasis on commonly occurring negative samples, potentially culminating in overfitting to their respective relationships. This phenomenon could manifest as a diminished performance when the model encounters unseen data, as it overly concentrates on assimilating information from familiar instances while neglecting relationships among other data points within the dataset. Furthermore, when η exceeds an optimal range, the model may disproportionately rely on a subset of data, thereby disregarding the broader characteristics of the dataset’s distribution and consequently compromising its generalization capabilities.

Hence, both suboptimal values of η, below or above 0.01, can be attributed to the model’s inability to effectively balance the emphasis placed on crucial samples and to mitigate overfitting. Consequently, when selecting values for η, it is imperative to carefully consider achieving a balance in the model’s attention across diverse samples and preventing overfitting to known data instances. Such meticulous consideration enhances the model’s capacity to generalize and augments its predictive performance.

## 4. Discussion

The experimental findings of this study demonstrate a significant improvement in predicting biomass from images when compared to traditional regression methods such as SCDR and the BioUMixer network. Furthermore, the appropriate selection of hyperparameters positively influences the outcomes of deep regression. In contrast to analogous regression methods like ConR [41] and RNC [40], SCDR incorporates the similarity between image features into the calculation of pairwise affinities. This integration allows SCDR to concurrently consider both types of information, resulting in more accurate and comprehensive affinity calculations and reducing interference from erroneous label pairs. The experimental results underscore the superiority of SCDR over ConR and RNC methods.

In similar endeavors targeting biomass prediction tasks, both of Albert’s studies have focused on forecasting the Above-Ground Biomass of pasture grass. Leveraging the Irish dry herbage mass dataset (the dataset is not publicly available), Albert embarked on two distinct initiatives. In the initial endeavor, Albert [58] adopted ResNet50 as the foundational neural network, merely modifying the ultimate output prediction head to forecast the herbage biomass. This approach yielded a peak performance with az *HRMSE* (*RMSE* in this paper) of 92.69. Subsequently, in the subsequent project, Albert [59] proposed a semi-supervised learning methodology in conjunction with high-resolution enhancement of drone imagery to estimate biomass, culminating in an improved *HRMSE* of 85.7. Comparative analysis of these two endeavors underscores the fundamental contributions of the present study. The proposed SCDR methodology addresses, at its core, the issue of relative sequence disparities between images and labels in regression tasks, offering a holistic resolution. Furthermore, the introduced BioUMixer, in contrast to conventional networks like ResNet, demonstrates superior feature extraction capabilities, rendering it better suited for biomass prediction tasks.

Nevertheless, this study does present certain limitations. For instance, the BioUMixer network exhibits a slightly complex structure and imposes a considerable computational burden. While SCDR accounts for image feature similarity, it also introduces additional computational overhead. The applicability of SCDR to non-image data remains uncertain and warrants further investigation. Potential solutions to address these limitations may involve the adoption of lightweight network architectures and the optimization of affinity and similarity calculation methods within SCDR to alleviate computational burdens.

In the future, we intend to explore enhancements to SCDR for non-image modalities, such as point cloud data, and strive to refine it into a modality-agnostic deep regression method.

## 5. Conclusions

This study is dedicated to the task of predicting aboveground crop biomass through image data. To address this challenge, we introduce the SCDR for the first time. This learning paradigm incorporates supervised contrastive learning into deep regression problems, innovatively considering labels and sample features when calculating sample pair forces. This innovation leads to a more ordered arrangement of sample labels in downstream regression tasks. Furthermore, we introduce the U-Like Hierarchical Residual Fusion Network (BioUMixer), a network designed for feature extraction from biomass image data, enhancing information exchange and fusion while comprehensively considering both global and local information features in images. Finally, we create a novel biomass prediction dataset named “Pepper_Biomass” and validate the effectiveness of our proposed methods and models on this dataset and the publicly available GrassClover dataset. Evaluation results on the Pepper_Biomass dataset yield *RMSE* = 252.18, *MAE* = 201.98, and *MAPE* = 0.107, while on the GrassClover dataset, the results are *RMSE* = 47.92, *MAE* = 31.74, and *MAPE* = 0.192. Future research directions will include considering different modal data generated from various sensors to enhance the accuracy of biomass prediction. Additionally, we plan to explore joint predictions of biomass by combining multimodal data.

## Figures and Tables

**Table 1:** Acquisition details of the Pepper_Biomass dataset.

Timing	September 2023	October 2023
Location	China, Xinjiang, Shawan City 84°57′ E, 43°29′ N	China, Xinjiang, Yanqi County 86°44′ E, 42°20′ N
Camera equipment	DJI drones	Smartphone
Chili varieties	Line pepper (Capsicum annuum var. longum)	Chili pepper (Capsicum annuum)
Delineation of subregions	1–20	21–100
Sub-area area	3 m^2^	1.5 m^2^
Acquisition method	Hand-picked	Hand-picked
timing	September 2023	October 2023
Location	China, Xinjiang, Shawan City 84°57′ E, 43°29′ N	China, Xinjiang, Yanqi County 86°44′ E, 42°20′ N
Camera equipment	DJI drones	Smartphone
Delineation of subregions	1–20	21–100
Sub-area area	3 m^2^	1.5 m^2^
Acquisition method	Hand-picked and sickle harvesting	Hand-picked and sickle harvesting

**Table 2:** Specific parameters of the filming equipment.

Capturing Devices	Color Depth	Image Size	Spatial Resolution
DJI	14-bit	5472 × 3078 pixels	10 cm^2^
Smart Phone	10-bit	3000 × 3000 pixels	16 cm^2^

**Table 3:** The performance of various models and regression losses in predicting pepper biomass on the dataset is demonstrated.

Metrics	*RMSE*				*MAE*				*MAPE*			
Model/Loss	Focal_L1	RNC	ConR	SCDR_Loss	Focal_L1	RNC	ConR	SCDR_Loss	Focal_L1	RNC	ConR	SCDR_Loss
ResNet50	372.15	335.12	326.44	293.08	319.24	279.69	258.84	236.51	0.184	0.147	0.137	0.125
EifficientNetV2	356.35	320.5	295.93	290.03	293.95	256.86	234.39	231.06	0.172	0.131	0.123	0.122
MLP_Mixer	366.15	325.21	315.23	294.29	298.73	272.87	255.71	236.12	0.179	0.146	0.135	0.126
ViT	376.72	366.01	346.78	328.23	313.38	304.38	284.61	266.38	0.182	0.162	0.154	0.14
DaViT	428.23	427.01	429.17	429.21	382.96	385.59	388.17	387.93	0.219	0.198	0.197	0.197
EfficientViT	348.25	314.49	302.31	295.79	297.32	258.69	241.89	241.53	0.158	0.135	0.123	0.128
Swin Transformers	435.53	435.59	384.51	352.53	388.23	388.08	318.82	295.11	0.203	0.198	0.172	0.15
ConvNeXtV2	433.51	326.06	321.81	301.95	381.8	265.73	260.17	252.16	0.216	0.142	0.138	0.132
BioUMixer	316.03	302.39	286.07	252.18	262.66	250.67	236.34	201.98	0.136	0.127	0.121	0.107

**Table 4:** Showcases the biomass prediction performance of different models on the GrassClover dataset.

Metrics	*RMSE*				*MAE*				*MAPE*			
Model/Loss	Focal_L1	RNC	ConR	SCDR_Loss	Focal_L1	RNC	ConR	SCDR_Loss	Focal_L1	RNC	ConR	SCDR_Loss
ResNet50	93.21	78.84	61.73	53.90	65.31	56.21	40.32	36.16	0.468	0.313	0.268	0.236
EifficientNetV2	89.13	77.29	58.63	48.97	66.67	53.49	42.16	37.4	0.422	0.357	0.297	0.275
MLP_Mixer	122.049	115.89	108.78	85.78	69.77	67.84	64.06	53.64	0.417	0.407	0.377	0.343
ViT	133.18	128.64	110.78	105.91	85.39	85.14	76.93	75.46	0.468	0.476	0.44	0.426
DaViT	137.5	136.73	137.65	138.05	90.53	90.82	90.58	90.54	0.501	0.492	0.493	0.487
EfficientViT	97.99	81.35	70.67	59.32	69.14	62.37	46.7	38.6	0.461	0.398	0.301	0.27
Swin Transformers	146.07	145.94	139.53	137.49	90.53	90.54	91.41	90.58	0.505	0.498	0.507	0.489
ConvNeXtV2	143.09	140.13	137.80	127.5	91.20	90.09	86.15	80.75	0.528	0.525	0.487	0.466
BioUMixer	86.32	71.54	56.97	47.92	61.39	52.98	41.49	31.74	0.381	0.351	0.272	0.192

**Table 5:** Ablation experiments on different modules of BioUMixer.

Settings		Case1	Case2	Case3	Case4	Ours
U-Like Hierarchical Residual		×	×	✓	✓	✓
Feature_block		×	✓	×	✓	✓
Bio_block		×	✓	✓	×	✓
Pepper_biomass	*RMSE*	414.78	316.85	283.23	290.85	252.18
	*MAE*	366.38	260.24	233.76	240.75	201.98
	*MAPE*	0.192	0.136	0.121	0.125	0.107
GrassClover	*RMSE*	130.78	98.17	71.06	64.99	47.92
	*MAE*	89.66	70.98	42.27	40.11	31.74
	*MAPE*	0.485	0.454	0.247	0.235	0.192

“×” indicates the removal of the module, and “✓” indicates the retention of the module.

**Table 6:** *β* Parametric ablation experiments.

*β*	*RMSE*	*MAE*	*MAPE*
0.1	288.86	238.02	0.122
0.5	295.93	244.72	0.128
1	304.51	251.63	0.131
2	292.58	241.02	0.127
3	252.18	201.98	0.107
4	268.51	214.83	0.114
5	265.14	215.54	0.112
6	271.04	227.42	0.123

**Table 7:** *τ* Parametric ablation experiments.

*τ*	*RMSE*	*MAE*	*MAPE*
0.1	301.85	254.71	0.132
0.2	252.18	201.98	0.107
0.3	277.68	226.02	0.12
0.5	267.04	220.58	0.117
1	260.19	214.95	0.112
2	278.53	232.29	0.123
5	280.86	226.73	0.121

## Data Availability

GrassClover: https://vision.eng.au.dk/grass-clover-dataset/ (accessed on 28 February 2024). Pepper_Biomass: https://zenodo.org/records/10939932 (accessed on 8 April 2024). Our SCDR and BioUMixer Model’s Code: https://zenodo.org/records/10935913 (accessed on 8 April 2024).
